# Editorial: Social stratification and social inequality in East Asia

**DOI:** 10.3389/fsoc.2026.1934826

**Published:** 2026-07-31

**Authors:** Yoshimichi Sato, Shin Arita, Chin-fen Chang, Chunling Li, Tai Lok Lui, Kwang-Yeong Shin

**Affiliations:** 1 Faculty of Humanities, Kyoto University of Advanced Science, Kyoto, Japan; 2 Institute of Social Science, The University of Tokyo, Tokyo, Japan; 3 Institute of Sociology, Academia Sinica, Taipei, Taiwan; 4 Institute of Sociology, Chinese Academy of Social Science, Beijing, China; 5 The Education University of Hong Kong, Hong Kong, Hong Kong SAR, China; 6 Department of Sociology, Chung-Ang University, Seoul, Republic of Korea

**Keywords:** social stratification, social inequality, East Asia, local culture, local history, local institutions

Social inequality is not created in a vacuum. It emerges within social contexts shaped by local histories, cultures, and institutions. This premise provides the theoretical foundation for this Research Topic. Research on social stratification and social inequality examines how valued resources—especially education, occupation, income, and wealth—are unequally distributed across social groups. The field has long examined two broad forms: inequality of opportunity (e.g., Featherman and Hauser, 1978) and inequality of outcomes (e.g., Piketty, 2013).

Inequality of educational opportunity refers to class differences in educational attainment. Children from advantaged backgrounds, for example, are more likely to enter higher education than those from disadvantaged backgrounds. Inequality of occupational opportunity concerns the extent to which first jobs are shaped by class origin and education, and how later positions are influenced by these earlier advantages and disadvantages. Income and wealth are often treated as outcomes, yet opportunities to acquire them are also conditioned by family background, education, first job, and current occupation.

To investigate these processes, scholars apply statistical models—including structural equation, log-linear, and log-multiplicative models—to empirical data. One influential example is the constant flux hypothesis associated with Erikson and Goldthorpe (1992), which argues that, in industrial societies, absolute mobility rates may vary with changes in occupational structure, while the relative association between class origins and destinations—that is, the pattern of social fluidity—remains comparatively stable.

This Research Topic proceeds from the view that the causal forces shaping opportunities and outcomes are mediated by local institutions, cultures, and histories. These factors may either mitigate or exacerbate inequality. Scholarships, for instance, may raise college entry rates among children from disadvantaged families and thereby reduce educational inequality. By contrast, segmented labor markets may maintain or intensify inequalities between employees in protected sectors and workers outside them.

East Asian societies provide an important setting for examining these issues. The region has experienced rapid economic development, educational expansion, and demographic transformation. It has also seen profound changes in labor markets, welfare policies, and family systems. Despite these shared experiences, East Asian societies exhibit considerable diversity in their political institutions, welfare regimes, labor market regulations, and occupational transformations. These distinct local factors have shaped unique patterns of social stratification and inequality that differ from those in Western societies. This Research Topic brings together six articles that share this theoretical orientation.

Zhanget al.'s article, “*Trap versus trade-off: Cohort evidence on overeducation, income returns, and group disparities after China's higher education expansion*,” examines how China's 1999 higher education expansion affected inequality. Although the policy was designed to meet the growing demand for college-educated labor, it also generated mismatches between educational attainment and job requirements. Using a hierarchical age-period-cohort model and nineteen cohorts from the Chinese General Social Survey, the authors show that the consequences of expansion varied across social groups. They call for targeted, group-sensitive interventions to address the unequal risks and opportunities created by educational expansion.

Yuan et al.'s article, “*Compulsory education law and intergenerational income mobility in China*,” turns to compulsory education. Although compulsory education is expected to reduce educational inequality, its effects require empirical verification. Using China Household Income Project data and a difference-in-differences design, the authors find that China's compulsory education law significantly reduced the intergenerational income correlation between parents and children, thereby increasing income mobility. They also show, however, that its effects varied by educational level and social group.

Chen et al.'s article, “*Cumulative effects of unemployment on health in midlife: The buffering effect of social participation*,” shifts attention to the labor market and health. Using longitudinal data from the China Health and Retirement Longitudinal Study, fixed-effects models, and factor analysis, the authors examine the cumulative impact of unemployment on the health of middle-aged and older adults, and the moderating role of social participation. They find that unemployment has significant adverse health effects, especially for men, and that unemployment duration affects health differently by gender. Informal social participation reduces these negative effects, whereas formal participation does not.

Imai's article, “'*Industrial citizenship' and social inequality in Japan: The dynamics of contract and status in shaping inequalities*,” explains how industrial citizenship has generated inequality in Japan. The paper traces the development of industrial citizenship into company citizenship and shows how inequality has been structured not primarily by class, but by firm size, gender, and employment status. In Japan, company affiliation may be as important as class location. The article thus offers an alternative to class-centered frameworks by emphasizing how historically contingent configurations of status and contract reproduce inequality.

Ishida's article, “*Transnational career advantages: Earnings growth of Japanese self-initiated expatriates*,” focuses on workers outside Japanese company citizenship: self-initiated expatriates. Using longitudinal survey data from the ADIOS-J project and JLPS dataset, the paper tests whether these workers experience higher earnings growth than comparable workers in Japan and whether they face economic penalties upon return. The findings support the first hypothesis for self-initiated expatriates employed in multinational companies. Returning to Japan itself does not penalize returnees; job characteristics and pre-migration conditions shape post-return outcomes.

Kim et al.'s article, “*Infertility perceptions and associated factors among Korean undergraduate students: A cross-sectional study*,” examines how Korean undergraduates perceive infertility and what shapes these perceptions. In South Korea, infertility has long been interpreted through sociocultural contexts that emphasize marriage, parenthood, and gendered expectations of childbearing. Yet infertility rates have been increasing, creating a gap between traditional expectations and social reality. The authors find that stronger endorsement of traditional gender roles and family-oriented values that prioritize childbearing is associated with more negative perceptions of infertility. They therefore underline the importance of infertility education.

Taken together, the papers in this Research Topic demonstrate that social inequality in East Asia cannot be understood without close attention to local cultures, histories, and institutions. We hope readers will find that this perspective also illuminates the study of inequality beyond East Asia—in societies East and West, South and North.
